# BM-BC: a Bayesian method of base calling for Solexa sequence data

**DOI:** 10.1186/1471-2105-13-S13-S6

**Published:** 2012-08-24

**Authors:** Yuan Ji, Riten Mitra, Fernando Quintana, Alejandro Jara, Peter Mueller, Ping Liu, Yue Lu, Shoudan Liang

**Affiliations:** 1 Center for Clinical and Research Informatics, Northshore University HealthSystem, Evanston, IL 60091, USA; 2 ICES, University of Texas at Austin, Austin, TX 78705, USA; 3 Department of Statistics, Pontificia Universidad Católica de Chile, Casilla 306, Correo 22, Santiago, Chile; 4 Department of Mathematics, The University of Texas at Austin, Austin, TX 78705, USA; 5 Abbott Molecular Inc., Des Plaines, IL 60018, USA; 6 Department of Leukamia, The University of Texas, M. D. Anderson Cancer Center, Houston, TX 77030, USA; 7 Department of Bioinformatics & Computational Biology, The University of Texas, M. D. Anderson Cancer Center, Houston, TX 77030, USA

## Abstract

Base calling is a critical step in the Solexa next-generation sequencing procedure. It compares the position-specific intensity measurements that reflect the signal strength of four possible bases (A, C, G, T) at each genomic position, and outputs estimates of the true sequences for short reads of DNA or RNA. We present a Bayesian method of base calling, BM-BC, for Solexa-GA sequencing data. The Bayesian method builds on a hierarchical model that accounts for three sources of noise in the data, which are known to affect the accuracy of the base calls: *fading*, *phasing*, and *cross-talk between channels*. We show that the new method improves the precision of base calling compared with currently leading methods. Furthermore, the proposed method provides a probability score that measures the confidence of each base call. This probability score can be used to estimate the false discovery rate of the base calling or to rank the precision of the estimated DNA sequences, which in turn can be useful for downstream analysis such as sequence alignment.

## Introduction

Next generation sequencing (NGS) such as Solexa sequencing (http://www.illumina.com) is a powerful tool producing massive sequences of short reads. It is considered the “digital” version of the classic microarray technology because in principle it measures the exact number of gene copies rather than relative abundances. NGS can be used for studies of sequence variations in genomes ([1,2]), protein-DNA interactions ([3,4]), transcriptome analysis ([5-7]), and *de novo* genome assembly [8]. The full potential of the technology is still being explored as quantitative researchers try to find efficient ways to streamline the sample processing and model the processed data.

**Figure 1:**
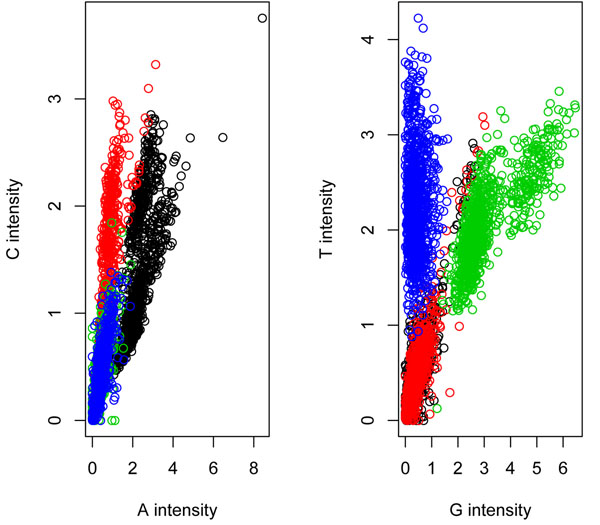
**Scatter plot.** The panel shows the scatter plots of the A-C and G-T pairs, constructed from the raw data alone. The y axis and the x axis in the left panel represent the C and A channels respectively. Similarly, the y and the x axes in the right panel denotes the T and G channels. The top panel consists of smoothed density plots of A intensities versus C intensities, and G intensities versus T intensities. The four colors in the figures of the bottom panel represent the estimated base calls from the proposed BM-BC method: black- A, red - C-green G, blue-T. The intensity values shown in the figure are normalized by subtracting from the overall minimum intensity and then dividing by the standard deviation.

Many challenges remain in processing NGS data. We consider one of the important problems, namely base calling. Base calling refers to the estimation of the true sequences of DNA or RNA based on the intensity scores measuring the signal strength of four nucleotides, A, C, G, and T. One of the most popular NGS technology is the Solexa/Illumina sequencing, in which intensity data from a standard run consist of millions of intensity measurements for the four bases of short reads spanning across the genome. For each short read, the measurements of their intensities are stored in an *I* × 4 matrix, where *I* is the length of the read (e.g., *I* = 36). Such a matrix corresponds to a *colony.* The positions *i* = 1, ..., *I* in the short read are sequenced in *cycles.* As a result, each row of the colony matrix contains measurements from a cycle in the experiment in which the sequence of a single base is synthesized. At each cycle, all four nucleotides (A, C, G, and T) labeled with four different fluorescent dyes are probed, thus producing a quadruple vector of fluorescent intensity scores. Figure 1 plots the A intensities versus the C intensities (top left panel) and the G intensities versus the T intensities (top right panel) for 1,000 arbitrarily chosen colonies. The four colors used in the bottom two panels represent the estimated base calls from the proposed BM-BC method. Figure 1 exhibits two main features. First, the A and C intensities are highly correlated as are the G and T intensities, which is known as the “cross talk” between channels [9]. Second, when the A or C intensity is large, both the G and T intensities are small; similarly, when G or T is large, both A and C are small.

In summary, the final data are millions of quadruple vectors. Each vector contains four continuous scores that represent the fluorescent intensities of nucleotides A, C, G, and T. Using these data, our task is to estimate the sequence of each short read.

We acknowledge that the proposed method in this paper deals with the data from Solexa genome analyzer. New sequencing technologies have been developed by Solexa/Illumina, such as the HiSeq series. However, numerous data sets have already been generated using the genome analyzer, which need to be properly analyzed. We believe that our proposed base-calling approach will contribute to the analysis of the existing data and also future data from experiments that still use the genome analyzer for sequencing. To our knowledge, a few methods for base calling are available in the literature. Most researchers use the default procedure, Bustard, built into the commercial software of the Illumina Genome Analyzer. The procedure yields an estimated base for each cycle along with a quality score called fast-q. The fast-q score measures the most likely base intensity relative to the three other intensities on a logarithmic scale from –5 to 40. In practice, DNA tags with small fast-q scores are discarded in Solexa base calling. A more recent statistical method of base calling is by [10], who considered a variety of issues in the sequencing data including the base calling. Other works include [11,12]. A recent addition to this group of methods is Ibis (Improved Base Calling for Genome Sequence Analyzer) [13]. Ibis applies multiclass Support Vector Machines to raw cluster intensities. The model is trained from data obtained from a reference genome.

In this paper, we propose a model-based Bayesian method of base-calling (BM-BC) for Solexa sequencing data. The BM-BC method presents a hierarchical model that applies a probabilistic-based inference for base calling. The estimation of model parameters is computed via Markov chain Monte Carlo (MCMC) simulations and the posterior samples are used to compute the probability that each base is A, C, G, or T. These posterior probabilities are used to estimate the true DNA sequences, to rank the base calls, and to compute the false discovery rates (FDR). The remainder of this paper is organized as follows: The Methodology section presents a probability model for base calling, and the posterior inference procedure. The section on Numerical examples presents the base-calling results for a Solexa sequencing data set using the BM-BC method and three other methods as comparison. The Discussion sections ends the paper.

## Methodology

**Figure 2:**
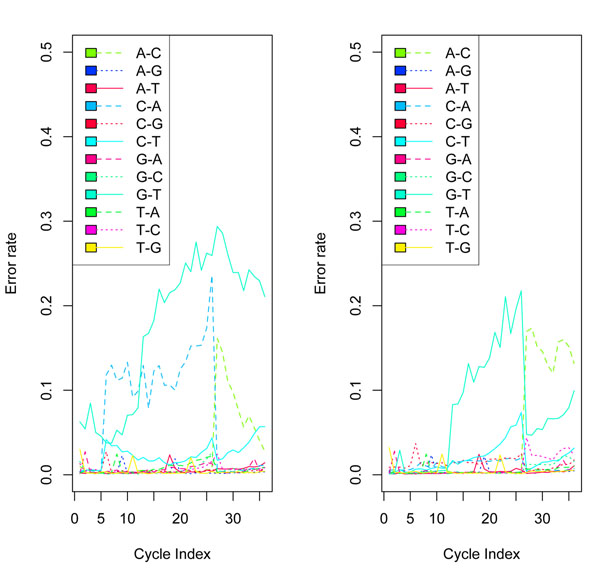
**Error rates for a random subsample of 1000 clusters**. (Colored figure) The error rate for each cycle. The error rate of the Solexa calls has a large increase after cycle 26, while the error rates of the BM-BC, B-I, and Rolexa calls increase gradually over the cycles.

To start, we introduce the three known sources of noise in the Solexa data that motivated the proposed probability models. The first type of noise is called *fading* (see e.g., [10]), which refers to a decay in the intensity as a function of cycle number. That is, for a colony, as the cycle number increases, the intensity measurement decreases. This is usually caused by material loss during the sequencing process. The second source is *phasing*, a well-known source of noise in Solexa sequencers that use cyclic reversible termination (CRT) ([14,15]). Basically, errors in the CRT cause stochastic failures in base-binding that is supposed to incorporate only one nucleotide per cycle. Instead, the errors may lead to incorporation of none or more than one nucleotide in one cycle, thus increasing the noise in the signal output for down-stream cycles. As a result, the precision of base calling drops as the cycle number increases (see Figure 2). The third important source of noise is a fluorophore *cross talk* between channels A and C, and channels G and T. The cross talk induces high correlations between A intensities and C intensities, and between G intensities and T intensities (see Figure 1). There are many factors that contribute to cross-talk between channels, one of them being an overlap in the wavelengths of the dye schemes used to mark different nucleotides.

Other important systematic biases also affect the accuracy of base calling. For a discussion, see [14,15]. However, these biases can be removed or reduced using standard statistical techniques. We assume that these biases have been removed and now the goal is to model the intensity scores.

### Hierarchical models

We first consider models for sequence data of a single colony, i.e., measurements corresponding to a short read, with say *I* = 36 bases. Let ***y*** = {***y***_1_, *…*, ***y***_36_} represent the 36 quadruplets of nucleotide intensity measurements, where ***y****_i_* = (*y_i_*_1_, *y_i_*_2_, *y_i_*_3_, *y_i_*_4_)′ is the 4 × 1 vector for cycle *i*, respectively representing the intensities of four nucleotides, A, C, G, and T at location *i* of the short reads. Therefore, strong signals are indicated by large positive values of *y_ij_.* Because for each cycle only one true nucleotide is present, ideally only one of the four *y_ij_*’s should be positive and the remaining three should be zero. In the presence of noise, this is not the case. First, due to channel *cross-talk*, *y_i_*_1_ and *y_i_*_2_ are positively correlated, as are *y_i_*_3_ and *y_i_*_4_. Second, because of *fading*, the intensities decay over cycles; that is, for later cycles, the values of ***y****_i_*’s are smaller on average. Last, when *phasing* is present, the intensity scores at cycle *i* depend on the ones at cycle (*i* – 1).

Let *k_i_* ∈ {1, 2, 3,4} indicates the true base of cycle *i*, where {1, 2, 3, 4} correspond to {A, C, G, T}. The main feature of the sampling model for ***y****_i_* is given by an auto-regression consisting of a mixture of four multivariate normal distributions, with each normal distribution describing the case when the true base is one of {A, C, G, T}. Specifically, letting MVN_4_(***µ***, Σ) denote a 4-dimensional normal with mean vector ***µ*** and covariance matrix Σ, we assume that for *i* = 2, …, 36,(1)

and(2)

where *I_j_*’s are four indicator functions *Ind*(·) that truncate the multivariate normal. Here, . These indicators reflect the prior belief that the true base should have the largest intensity. Models (2) and (2) attempt to account for three sources of noise in the data. Specifically, due to fading, the intensity signals weaken as the cycle indicator *i* gets larger. Therefore, we include the exponential factor exp(–*β · i^λ^*) to describe the decay of the mean signal. Note that we specify an exponent *λ* to allow for more flexibility. For the phasing, we add a term *α · y_i_*_–1_,*_j_* to the mean of the multivariate normal (thus autoregressive), i.e., the intensity of the current cycle *i* depends on the intensity of the previous cycle (*i –* 1) for *i* ≥ 2.

The cross talk is accounted for by constructing appropriate priors for ***μ****_j_*’s, as described next. We assume that the mean intensities when the true base is A, C, G, or T are given by

When the true base is A (i.e., *j* = 1), the intensities at channels A and C are modeled by *µ*_11_ and *µ*_12_ while the intensities at channels T and G will be close to zero, parametrized as *ε*_11_ and *ε*_12_. In addition, the mean intensity *µ*_11_ at channel A should be larger than *µ*_12_ at channel C. Therefore, the prior for ***µ***_1_ is given by(3)

We use a log N(0,1) prior for *µ*_11_*.* Here, *g*_1_ accounts for the cross talk from channel C to channel A. We assign a *beta*(1, 1) as its prior. For *g_ε_*_1_ and *g_ε_*_2_, we use *beta*(2,10) to reflect our strong belief that the intensities at channels G and T are much smaller than the intensity at channel A. We have tried other beta priors *beta*(*a*, *b*) with *a* ≪ *b* and obtained similar results in base calling.

The model is completed by specifying the discrete uniform prior for *k_i_*, i.e., *Pr*(*k_i_* = *j*) = 1/4 for *j* = 1, 2, 3, 4, a *beta*(1, 1) prior for *λ*, *α*, and *β*, and an inverse Wishart(*diag*(1, 4), 6) prior for ∑*_j_*, where *diag*(1, 4) is the 4 × 4 identity matrix.

The models above are built for one colony of sequencing data. With multiple colonies, we use ***y****_ic_* = (*y*_*ic*,1_, …, *y*_*ic*,4_) to denote the quadruple intensities of cycle *i* in colony *c*, and *k_ic_* to represent the latent indicator of the true base of cycle *i* in colony *c*. The models for ***y****_ic_* are the same as in (2) and (2), with *y_ic_* and *k_ic_* replacing ***y****_i_* and *k_i_*. The priors for *k_ic_*, ***µ****_j_, λ, α*, *β*, and ∑*_j_* remain unchanged. Since ***y****_ic_*’s are conditionally independent, the joint likelihood for all the data is simply the product of the likelihood function for each ***y****_ic_*. For simplicity, the mathematical expression of the models is omitted.

### Posterior inference

Inference is carried out via MCMC simulations. The probability models are coded in C (now included in an R package). The MCMC simulations output provides Monte Carlo posterior samples of all the parameters from the joint posterior distribution. These samples can be used to perform posterior inference. For example, we obtain random samples of *k_ic_* from its marginal posterior, denoted as , where *B* is the number of MCMC samples. We can compute(4)

as the posterior probability that the *i*th cycle in colony *c* has a true base of A, C, G, or T, respectively. These samples can be used to perform base calling. Specifically, the Bayesian base call corresponds to the nucleotide with the largest posterior probability in its cycle. That is, we assign base A, C, G, or T to cycle *i* in colony *c* if *s_ic_* equals 1, 2, 3, or 4, where . In addition, one can assess the accuracy of the proposed method by computing an estimated Bayesian FDR ([16,17]) using the *ξ*’s. We will demonstrate this feature with a concrete example in the next section.

## Numerical examples

We compared the performance of the BM-BC method with currently leading methods, including the Solexa Bustard, the Rolexa method [11], and the B-I method [10].

### Data

We obtained Solexa DNA sequencing data from the control lane for a bacteria phage. This is part of the standard Solexa protocol. To illustrate the performance of base calling methods, we randomly selected three subsets, with each containing 1,000 colonies of the sequence data.

The control lane sequences the genome of an enterobacteria phage, phiX174, which is composed of 5,386 bases of single stranded DNA sequences and has no polymorphism. DNA preparation follows Illumina Control DNA library protocol (Illumina Cat. No CT-901-1001). DNA are broken to a size of 200 nucleotides and are subject to 18 cycles of polymerase chain reaction (PCR) amplification before the generation of DNA colonies by single molecule PCR. The sequences of DNA colonies are probed by 36 cycles of sequencing by synthesis.

Each DNA read is compared to the entire phage genome of 5,386 positions to search for the best matches. This is done using the Solexa software PhageAlign. After a tag is aligned to the phage genome, the matched sequence on the phage genome is considered to be the true sequence and any mismatched nucleotide is considered a sequencing error. The assignment of the true sequence is correct because 1) the phage genome contains no polymorphism and 2) the small genome size makes a mistaken sequence match over 36 nucleotides highly unlikely. Note that this is not the case for the human genome, where polymorphism occurs ([18]). Here, we treat the bases obtained from the above procedure as the “true” ones and compare the performance of base calling methods based on the deviation from these bases.

### Analysis with random subsets

We first applied all the methods to a small data set for illustration purpose. We then implemented the BM-BC method on a data set from the control lane of the Solexa sequencing, consisting of about 5 million short reads. We compare the following four base-calling methods using the phage sequencing data.

• Bustard from Solexa’s Genome Analyzer: this is the commercial software provided by Illumina. More detailed information about the Genome Analyzer can be found at http://www.illumina.com.

• Rolexa: this is a method building upon model-based clustering [11], which assumes that the quadruplets of intensities follow four-component univariate Gaussian mixture models. Instead of performing a full Bayesian inference using the joint posterior distribution, the Rolexa method applies the EM algorithm to obtain point estimates of the parameters.

• B-I: this is the intensity model proposed in Bravo and Irrizary (2010). The authors carefully examined potential noises in the intensity data and proposed a linear mixture model with different means given the indicator of true bases. They applied the EM algorithm to obtain the posterior probabilities of the true base calls. See [10].

• BM-BC: our proposed method.

We applied all four methods to the three random subsets of phage sequencing data, each with 1,000 colonies.

For the BM-BC method, we performed base calling using 100 colonies at a time. The Markov chains converged fast and mixed extremely well. We only needed to throw away 100 burn-in samples with a total of 600 iterations for every 100 colonies.

We compared the estimated bases from the four methods with the true bases. Table 1 shows the number of wrong calls for each of the four methods. The BM-BC method had the smallest number of wrong calls for two subsets and a close second for the third subset, in which the Rolexa yields the smallest number of wrong calls.

**Table 1:** Error rates for different methods under comparison

Data sets	Number of wrong calls (percentage)			
	
	BM-BC	Solexa	B-I	Rolexa
1	**1,340 (3.7%**)	1,455 (4.0%)	1,428 (4.0%)	1,601 (4.4%)
2	**1,354 (3.7%**)	1,514 (4.2%)	1,426 (4.0%)	1,432 (4.0%)
3	1,385 (3.8%)	1,438 (4.0%)	1,444 (4.0%)	**1,345 (3.7%**)

The number of wrong calls for the methods under comparison: the proposed BM-BC, Solexa calls from the Bustard method, the method in Bravo and Irizarry (2010) (B-I), and the Rolexa method. Three subsets of Solexa sequencing data for a bacterial phage were selected, each with 1,000 colonies. Each row contains the number of missed calls (out of 36,000) for a subset. The bold entry in each row indicates the method with the fewest wrong calls.

In Table 1, we used ACGT as the base calls for the Rolexa method. In the original paper by Rougemont et al. (2008), the authors focused on using the International Union of Pure and Applied Chemistry (IUPAC) symbols (http://www.bioinformatics.org/sms/iupac.html) as base calls. These symbols include not only ACGT, but other ambiguous calls that represent more than one base within ACGT. The authors stated that the IUPAC symbols gave the Rolexa better performance. For a fair comparison, we used the ACGT symbols for the Rolexa.

For ease of exposition, we now focus on the results of an arbitrary subset, data set 1 in Table 1. We computed the difference in the number of correct calls per colony between the BM-BC method and each of the other three methods.

We can see that the BM-BC method is more likely to make right calls for a given colony than the other three methods. In addition, in extreme cases the BM-BC method could make more than 20 more correct calls (out of a total of 36) than the other methods. In contrast, the largest number of more wrong calls the BM-BC method could make is only 6. Figure 2 compares the error rates by cycle, defined as the proportion of wrong calls for each cycle across all colonies. Interestingly, the error rate for the Solexa calls has a large increase after cycle 26. See Figure 5 for more results related to this. This seems to suggest that the Solexa base calling is more sensitive to the phasing noise in the data. In contrast, the error rates for the other three methods increase gradually over the cycles. Both BM-BC and Rolexa methods are also robust to phasing as it is specifically accounted for in the probability models. We can estimate the FDR based on the posterior probabilities *ξ*’s for base calls from the BM-BC method. Because we know the true bases, we can precisely compute the FDR of the BM-BC method. The idea is to treat  as the local FDR. We present the following algorithm for computing the FDR based on the true bases.

1. Let the true base be *t_ic_* for cycle *i* in colony *c*.

2. Compute ; then  is the local FDR denoting the posterior probability of making a wrong call.

3. Rank the pairs (*i*, *c*) according to the increasing values of .

4. Starting from the highest ranking pair (*i*, *c*) with the smallest , move down to the *G*th highest ranking pair. The estimated FDR is given by the sum of  for all *G* pairs divided by *G.*

**Figure 3:**
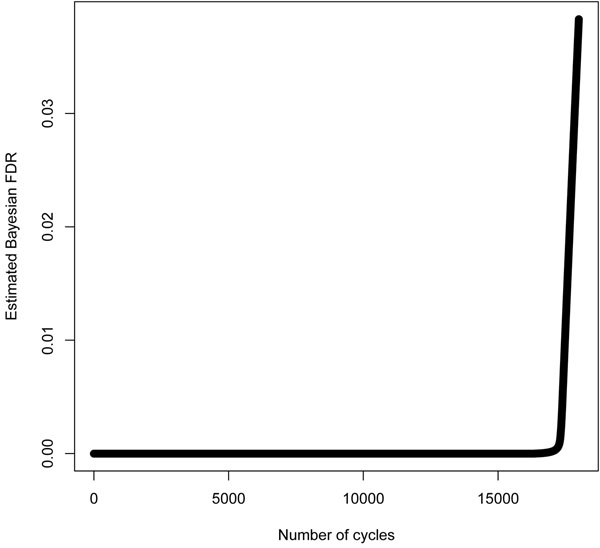
**FDR plot**. Bayesian FDR plot with 18,000 base calls under the BM-BC method.

Figure 3 plots the estimated FDR versus the number of calls (ranked based on increasing values of ). We can see that the FDR is controlled by 0.04. This seems to agree with the error rate in Table 1. In cases where we do not know the true base calls, we only need to replace *t_ic_* with , the estimated base call by the BM-BC, in the above FDR algorithm to estimate the Bayesian FDR. This new value will be smaller because the errors in *s_ic_* are not accounted for.

### Full data analysis

We implemented the BM-BC method on a data set consisting of 5,120,000 colonies. The data are from a control lane in a standard Solexa run, in which the true sequences are known. We first splitted the data into 8 equal parts, each comprising of 640,000 colonies. We then applied the BM-BC method to each of the eight subsets in parallel. The eight jobs were executed on an iMAC with 2.8 GHz Intel Core i7 and 16 GB of memory. It took about 4 hours to complete the computation. We have built an R package “BM-BC”, available to be downloaded from http://odin.mdacc.tmc.edu/~yuanj/soft.html

**Figure 4:**
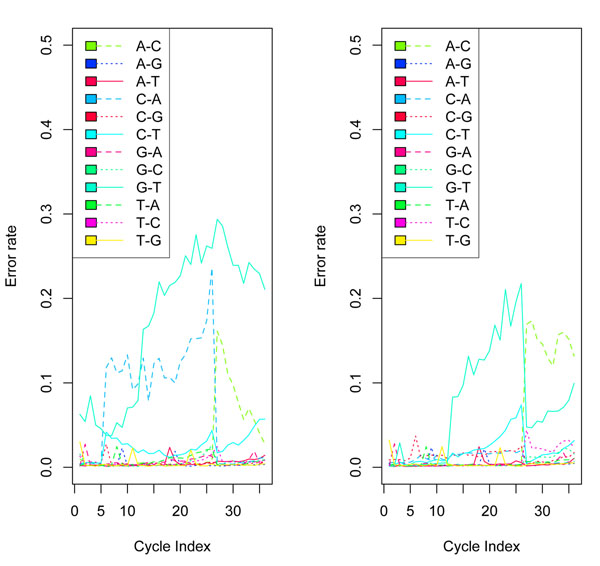
**Comparison with Bl method.** Comparison of Base errors per cycle for the BM-BC method (right panel) and the B-I method (left panel) in Bravo and Irizarry (2010) for a random subset of 50,000 colonies. The error rate of base calls is about 4.9% for the BM-BC and about 8.0% for the B-I method. The G-T substitution error curve (shown by a turquoise green solid line) and the A-C substitution curve (shown by a blue dotted line) dominates the other pairwise substitution rate in both the methods. However, clearly, the curves in the BM-BC are lower both in the absolute scales and in the rate of increase with cycles.

**Figure 5:**
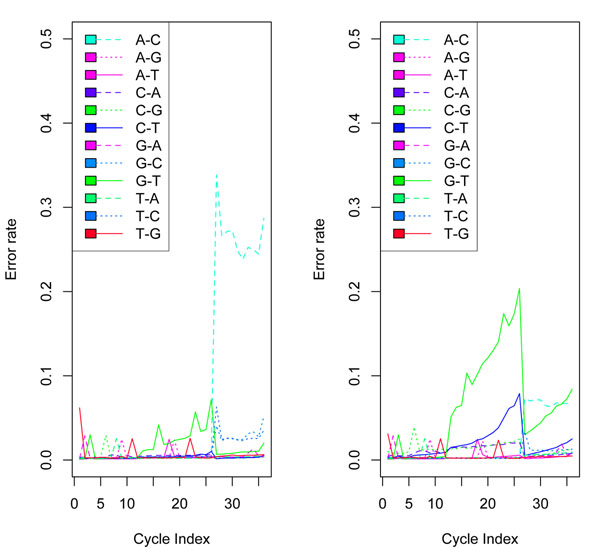
**Comparison with Solexa method.** (Colored figure) Base errors per cycle for the entire dataset based on the BM-BC (top panel) and the Bustard under Solexa sequencing (bottom panel). The plot further confirms that for the BM-BC method, there is no increase in base substitution errors with increasing cycle, a common problem in most basecalling methods. Also the major potential substitution errors, A-C and G-T substitutions have been accounted for quite well. For the Bustard method, there is a large increase in the error rates (after cycle 26, shown by the green dotted line) for A-C substitutions. Both methods yield an overall error rate of 4% in base calling.

We computed  as the posterior probability that the base of cycle *i* in colony *c* is *j*, for *j* = 1, 2, 3, or 4. The base call is , the base with the largest posterior probability. We found that almost all the largest posterior probabilities were greater than 0.95, thus implying that our model was able to predict most bases with high degrees of confidence. Since we knew the true sequences for the data, we compared our predicted calls to the true sequences. Table 2 cross-tabulates the comparison results. In Figure 4, we see that the B-I error curves, though showing no such drastic jumps, still fares poorly compared to the BM-BC method. For this dataset the B-I also has a larger overall error rate of 8% compared to that of BM-BC, which has an overall error rate of of 5%. Figure 5 plots the error rates by cycle for the BM-BC and Solexa methods using the entire dataset. Although the overall error rates for the BM-BC and the Solexa methods are comparable, the A-C substitution rate for the Solexa calls show a large increase after cycle 26.

**Table 2:** Basecall Matching Rates

		Predicted calls			
		A	C	G	T
	A	97.22	2.00	.3	.3
True calls	C	1.06	95.75	1.29	1.88
	G	.00	.00	92.89	6.33
	T	.00	.01	.00	98.52

Matching rates of Basecalls by percentages. The overall matching percentage is 96.24.

## Discussion

An important feature of the BM-BC method is that it yields marginal posterior probabilities of the four nucleotides for each base. This allows a full probability-based inference for base calling and subsequent analysis. For example, one can associate the posterior probability of the base call with the estimated base and use it as a quality control measure for downstream sequence alignment. Sequences mapped to a genome with overall high posterior probabilities are more reliable than those with lower probabilities.

We also compared our method with the Bayesian classifier BayesCall in [12]. The computation was slow compared to the other methods. The slow speed could be a potential shortcoming for its application to data from NGS platforms, typical consisting of about millions of clusters. Naive Bayes classifiers, on the other hand, suffer from the simplistic assumptions of independence which are grossly violated in datasets of these type. One important feature of BM-BC is that it does not require any prior learning for its application to GA-I data. However, unsupervised clustering is not always feasible for data from newer sequencing technologies. Ibis [13] specifically uses large training data sets to analyze GA-II control lanes. In addition, certain platforms possess unique features and need algorithms specially tailored to their specific requirements. Ibis, for example, is designed to model the features of bi-directional phasing and T accumulation which are present in GA-II. On the other hand, BM-BC is more suited towards addressing the issues of phasing, fading and cross talk that arise in the context of modeling GA-I data.

We acknowledge that there is a scope of improving the model by incorporating the error sources unique to the latest sequencing platforms.

## Competing interests

The authors declare that they have no competing interests.

## Authors' contributions

Conceived and designed the method: YJ FQ AJ SL. Performed the data analysis: YJ RM FQ AJ PL. Wrote the paper: YJ RM FQ PM YL SL.
